# Vibrational and stochastic resonances in driven nonlinear systems: part 2

**DOI:** 10.1098/rsta.2021.0003

**Published:** 2021-05-31

**Authors:** U. E. Vincent, P. V. E. McClintock, I. A. Khovanov, S. Rajasekar

**Affiliations:** ^1^ Department of Physical Sciences, Redeemer’s University, P.M.B. 230, Ede, Nigeria; ^2^ Department of Physics, Lancaster University, Lancaster LA1 4YB, UK; ^3^ School of Engineering, University of Warwick, Coventry, CV4 7AL, UK; ^4^ School of Physics, Bharathidasan University, Tiruchirappalli 620 024, Tamilnadu, India

**Keywords:** nonlinear systems, vibrational resonance, stochastic resonance, driven oscillators

## Abstract

Nonlinearity is ubiquitous in both natural and engineering systems. The resultant dynamics has emerged as a multidisciplinary field that has been very extensively investigated, due partly to the potential occurrence of nonlinear phenomena in all branches of sciences, engineering and medicine. Driving nonlinear systems with external excitations can yield a plethora of intriguing and important phenomena—one of the most prominent being that of resonance. In the presence of additional harmonic or stochastic excitation, two exotic forms of resonance can arise: vibrational resonance or stochastic resonance, respectively. Several promising state-of-the-art technologies that were not covered in part 2 of this theme issue are discussed here. They include *inter alia* the improvement of image quality, the design of machines and devices that exert vibrations on materials, the harvesting of energy from various forms of ambient vibration and control of aerodynamic instabilities. They form an important part of the theme issue as a whole, which is dedicated to an overview of vibrational and stochastic resonances in driven nonlinear systems.

This article is part of the theme issue ‘Vibrational and stochastic resonance in driven nonlinear systems (part 2)’.

## 1. Introduction

Nonlinear systems are abundant in natural and engineering systems. Their dynamics has since the early 1960s attracted research attention following the proposed Lorenz model of atmospheric convection that has now been universally embraced as a paradigm for a wide range of investigations [1]. The vigour of ongoing research activity on nonlinear systems can be attributed to the wide variety of intriguing properties that emerge and to its interdisciplinary importance underlying all natural sciences, engineering and medicine.

In many instances, nonlinear systems are subjected to external influences acting as driving forces, thus allowing for their classification into *undriven* and *driven* systems, where the driven class of systems are those under the influence of external forces. The driving force, may consist of either deterministic excitations (e.g. harmonic signals) or stochastic actions (i.e. noisy inputs) [2]. It may include multiple frequencies, the simplest form being a bi-harmonic/dual-frequency driving force, and is often engineered to act as a control input with the aim of achieving, e.g. the general improvement of system performance [3], amplification and detection of weak signal inputs [4–6], enhancement of operating conditions and efficiency [7,8], control of the ion mean flux and ion energy in plasmas due to electrical asymmetry effect (EAE) [9–11], control of aerodynamic instabilities [12], particle excitation in magnetic particle imaging (MPI) [13,14], design of logic gates [15] as well as the control and annihilation of chaos and coexisting attractors [16]. It has been established that these applications are connected to a plethora of dynamical phenomena induced by driving forces, yielding insights into a diversity of microscopic and macroscopic processes as enumerated in a very recent review by Vincent & Kolebaje [2]. Among the phenomenon associated with multi-frequency driving, vibrational and stochastic resonances form the focus of this theme issue. They are arguably the most prominent forms of nonlinear resonance to have been investigated in the last 20 years, probably on account of the number of potential applications and ease of implementation [17,18].

The phenomenon of resonance, its definitions and its occurrence in biological, chemical, physical, mechanical and engineering systems were discussed in the introductory article of part 1 of this theme issue [18]. The concepts of vibrational resonance (VR) and stochastic resonance (SR), their origin, similarities and differences [19] in the context of driving forces as well as their manifestations and applications [3,5,15,20] were all described there. Here, we again emphasize that, although nonlinear resonance in driven systems has its origin in physics and mathematics, it is the diverse applications in other disciplines that has motivated the theme issue. We anticipate that it will provide a platform for cross-fertilization of ideas, thereby enabling researchers and practitioners in widely separated areas to become aware of each other’s work—a feat no journal has achieved to date.

## 2. General content of part 2

The general content of the theme issue has been described explicitly in part 1 [18] and organized with papers in three broad domains: theory, methods and analysis; complex networks and experimental applications. While a few articles in part 1 presented analysis on position-dependent mass systems [21], delayed feedback-induced resonances in deformable potential system [22] and Duffing oscillator [23], and driven two-level quantum systems [24], part 2 assembles state-of-the-art, original contributions on VR and SR in some systems not covered by part 1. These include a Brownian particle confined in a structural cavity [25,26], coupled neuron-astrocyte [27] systems and neural networks [28,29]. Part 2 also presents articles in four application domains: image perception [6], energy harvesting [8], control of aerodynamic instability [12], and vibrational machines and devices [30].

More importantly, driven nonlinear systems often form elements of complex networks, so that the impacts of the collective dynamics arising from network interactions on the response to the driving forces of an individual system should not be overlooked. In this part, there are two papers on complex networks—one of these reviews the enhancement of weak signals in complex networks due to stochastic and vibrational resonances [28], while the second article considers the effects of different kinds of autapses in the chaotic response of a single neuron and a network [29].

In summary, part 2 consists of the following 10 articles including the present Introduction:

1. Introduction: Vibrational and stochastic resonances in driven nonlinear systems—part 1 by Vincent *et al.* [31].
2. Stochastic and vibrational resonance in complex networks of neurons, by Calim *et al.* [28].
3. Vibrational resonance in a neuron–astrocyte coupled model, by Calim *et al.* [27].
4. Impacts of autapse on chaotic resonance in single neurons and small-world neuronal networks, by Baysal *et al.* [29].
5. Characterizing stochastic resonance in a triple cavity, by Mei *et al.* [26].
6. Entropic stochastic resonance induced by a transverse driving force, by Du *et al.* [25].
7. On some applications of vibrational resonance on image perception: the role of the perturbation parameters, by Morfu *et al.* [6].
8. The response of a bistable energy harvester to different excitations: the harvesting efficiency and links with stochastic and vibrational resonance, by Khovanov [8].
9. Suppression of galloping oscillations by injecting a high-frequency excitation, by Alhadidi *et al.* [12].
10. Energy and frequency ripple in devices with inertial excitation of oscillations. by Blekhman *et al.* [30].

The paper by Calim *et al.* [28], immediately following this Introduction, presents an intriguing and comprehensive review of recent works on stochastic and vibrational resonance in simple and complex networks of neurons. These systems are widely known to exhibit resonances on all scales—microscopic, mesoscopic and macroscobic—thereby benefiting from resonance mechanisms in the various tasks that they perform, including the propagation of information via weak signals. The paper reviews different types of SR and VR measures as well as different network topological structures such as *Motifs*, *Small-world*, *Scale-free*, *Feed-forward* and *Modular* that could be periodically or stochastically driven into resonance states. Calim *et al.* [28] focus on weak signal detection via both SR and VR in complex neuronal networks, from experimental and theoretical perspectives, and provides a guide to approaches for analysing SR and VR as well as the necessary and sufficient conditions for their occurrence. This review article is then followed by a research paper by Calim *et al.* [27], reporting new findings on VR in neurons interacting with a special class of glial cells known as astrocytes which controls their microenvironment. It is well known that astrocytes can modulate the activity of neural circuits that generate motor rhythms via alternating excitability of cells, transmission capacity of synapses and their plasticity; and can provide a feedback mechanism with neurons and their synapse, thereby playing important roles in neuronal information processing. Calim *et al.* [27] report investigations into the effects of intracellular astrocyte dynamics on the weak signal detection performance, and show that the resultant effects of the neuron-astrocyte coupling on VR is the appearance of double resonance peaks, referred to as double vibrational resonance (DVR). These are attributed mainly to the slow, long-time variation in neuronal excitability that can be significantly influenced by astrocytic calcium oscillations when the astrocytic Ca^2+^ dynamics are appropriately adjusted.

Baysal *et al.* [29] examine the impact of an autapse on chaotic resonance (CR) in single neurons as well as on small-world neuronal networks. An autapse is a special kind of synaptic contact, either electrical or chemical, connecting the axon of a neuron onto its own dendrites. The authors show that CR, which occurs when a system responds to a weak signal under the influence of a chaotic driving force, can be increased significantly by means of appropriately chosen autaptic parameter values.

**Figure 1.**
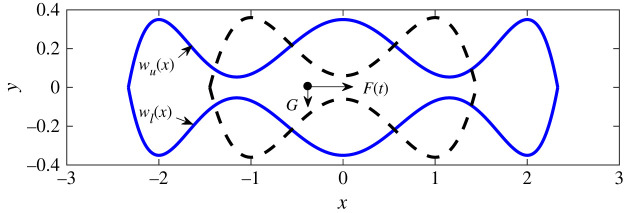
A schematic diagram illustrating the geometric boundary confinement of the motion of a archetypical Brownian particle in double (dotted lines) and triple (solid line) cavities. *F*(*t*) and *G* are applied forces on the particle. The upper and lower boundaries of the confinement channel are denoted by *w*_*u*_ (*x*) and *w*_*l*_ (*x*), respectively [26]. (Online version in colour.)

Owing to the existence of structural confinement in biological and soft condensed matter systems, the accessible phase space for a system is reduced and this also impacts on internal particle transport. Such confinement is often modelled by channels consisting of single, double, triple or even more periodic units and has been a subject of intense research on account of the entropic barriers arising from the uneven confinement boundaries. The problem of particle transport in confined structures have been broadly treated in different contexts, including the effect of confinement on SR. In the paper by Mei *et al.* [26], SR in a triple-cavity confined structure driven by Gaussian noise is analysed and characterized in the overdamped limit. Early studies of this problem were focused on double-cavity confined structures subjected to a constant force, acting in a direction transverse to a periodic force acting along the axis. A schematic diagram illustrating the geometric boundary confinement of the motion of an archetypical Brownian particle in double and triple cavities is shown in figure 1. Mei *et al.* [26] examine the similarities and differences of SR in both geometries and consider the differences between SR in a triple cavity from SR in a triple-well potential, as well as the influence on SR of boundary parameters and external forces. Finally, the work is extended to the case of fractional Gaussian noise to reflect some complicated situations, such as can be found in lipids diffusing in bilayers and tracers diffusing in living cells, where Gaussian noise would not be appropriate for the complete characterization of the random fluctuations. The authors conclude that the triple-confinement structure can induce a larger maximum in the spectral amplification.

Also on the topic of systems in confined channels, Du *et al.* [25] propose an alternative approach for controlling the dynamics of small-scale systems via *entropic stochastic resonance* (ESR). They report their investigations into the response of a Brownian particle moving within a dumbbell-shaped confinement, and driven by transverse driving force while preserving the system’s symmetry. The dumbbell-shape is similar in structure to the double (dashed lines) cavity shown in figure 1. The preservation of the system’s symmetry is in contrast with earlier studies of ESR in double cavities and in the absence of transverse periodic or static forcing [32,33]. Du *et al.* [25] emphasize that, when the boundary variations at the bottleneck are continuous, the transverse driving field induces entropy in the weak noise region, whereas high noise intensity eliminates the entropy trapping, leading to ESR. Furthermore, they show that ESR can conveniently be modulated by the transverse periodic force, thereby providing an alternative means for effective control of the dynamics of small-scale systems [32,33]. In a very recent paper, Du *et al.* [34] demonstrated, numerically and theoretically, the existence of VR for Brownian particles confined within an uneven boundary. The results in [25,26,34] coupled with the current burst of research activity on the dynamics of particles in confined channels clearly open new directions for investigating VR in confined structures.

In the application domain, Morfu *et al.* [6] review some applications of nonlinear resonances to image processing and visual perception, with specific emphasis on the role of noisy and high-frequency parametric perturbations, in the context of SR and VR. Morfu *et al.* [6] begin with a concise review of some general applications of nonlinear resonances in the field of image processing, image perception and signal dithering. They introduce a detector based on VR and SR formalism. They highlight the limitations of SR in image perception and describe a VR perturbation technique that can be employed to overcome these limitations of SR. They conclude that, in general, strategies based on VR could yield better results in certain applications where SR is used; and that their results should motivate further investigations of more complex detectors relevant to applications in image processing.

The harvesting of energy from various forms of ambient vibration has become a valuable techniques for remotely or locally powering the operation of miniature electronic devices, such as sensors and wireless communications. Relevant to the application domain, Khovanov [8] examines the response of an energy harvester to three kinds of excitation, focusing on its links with SR and VR in connection with the harvesting efficiency. By using a model bistable energy harvester system, comprised a mechanical structure (a beam), piezoelectric strips and an electrical circuit (load), Khovanov [8] explores the response of the harvester to *white noise*, *harmonic noise* with a narrow spectrum and *harmonic signals*, with respect to the efficiency of energy transfer as characterized by the power ratio. In the presence of a single input signal, it is shown that the underdamped dynamics of the harvester constitutes an important factor that impacts on the harvester’s energy transfer efficiency. However, the action of an additional low-frequency harmonic signal induces the phenomena of SR and VR, which manifest differently depending on the nature of the additional signal. Using a high-frequency harmonic signal, the coherence factor and power ratio depend on the underdamped dynamics (i.e. frequency), and their peaks are dependent on the harvester’s nonlinearity and bistability. On the other hand, the action of an additional stochastic signal ‘smooths’ the dynamics around each state, thereby weakening the impact of intra-well nonlinearity, so that bistability dominates. Khovanov [8] suggests that, in order to use a bistable design effectively, the dynamics of the velocity component should also demonstrate bistability, and that this could be achieved, for example, by using additional piezoelectric components to feedback some of the harvested electrical energy.

Alhadidi *et al.* [12] introduce a novel approach for suppressing galloping oscillations over a wide range of flow velocities that is capable of inducing them. The authors demonstrate, both theoretically and experimentally, that a high-frequency non-resonant base excitation can have this desirable effect. Galloping is a well-known phenomenon in aerodynamics, and was originally characterized by Den Hartog in the early twentieth century. It is a high amplitude periodic oscillation of elastic structures due to aeroelastic instabilities inciting oscillatory motion when they are subjected to an incident flow. Alhadidi *et al.* [12] discuss extensively the four prominent methods for suppressing galloping and their proposed new methodology based on injecting a strong high-frequency non-resonant excitation into the oscillator. The concept is motivated by existing theoretical and experimental approaches for altering the slow dynamics of nonlinear oscillators by injecting a strong high-frequency non-resonant excitation [35]. This kind of approach has been successfully employed in previous research for (i) stiffening a slow response, (ii) removal of discontinuities in non-smooth dynamical systems, (iii) shifting the static equilibrium point and (iv) stabilizing otherwise unstable equilibria of a nonlinear oscillator. Alhadidi *et al.* [12] demonstrate the proposed method and opine that its effectiveness is dependent on the profile of the aerodynamic lift force, which can differ for different bluff bodies, and can be improved by increasing the ratio between the base excitation amplitude and its frequency.

Finally, Blekhman *et al.* [30] examine the problems of machines that exert vibrational forces on materials. The authors focus on the theoretical and experimental analysis of energy and frequency ripple in vibrational devices consisting of softly vibration-isolated rigid bodies under the action of vibrations transmitted by means of inertial vibration exciters (unbalanced rotors driven into rotation by electric motors). By using a second-order approximation of the corresponding nonlinear equations, they show rigorously that there is a continuous exchange of energy between the rotor and the oscillating body, which is contrary to previous results obtained by use of the first-order approximations. Although the observed vibration effects might seem to portend harmful influence in, for instance, failures of synchronous and in-phase rotation of self-synchronizing vibrators, as found during the tests of a large vibration machine, the authors discuss some potential industrial applications in the design of new types of inertial vibration exciter and they propose a dynamical scheme for an exciter based on the VR phenomenon.

## 3. Summary and conclusion

The theme issue, which appears in two parts, focuses on the rapidly growing scientific subfield of VR and SR in nonlinear systems driven by dual-frequency forces, in which the second driving force could arise from the action of either a harmonic signal or stochastic signal. This second part contains 10 articles, addressing a range of interdisciplinary problems in physical and engineering systems as well as biological neural networks. While each of the articles presented comprehensive and insightful literature reviews that will enable readers, in particular non-specialists, to appreciate the presented concepts and results, two review articles are dedicated to complex networks [28] and image processing [6] that aggregates and reviews the state of the-art on VR and SR and their various experimental applications in these fields. This volume also emphasizes applications to machines and devices that exert vibrations on materials [30], the harvesting of energy from various forms of ambient vibration [8], and the control of aerodynamic instabilities by means of non-resonant base excitations [12]. As in part 1, part 2 also presents in concrete terms the scientific connections between VR and SR [6,8,28], thus providing a reference point and firm basis for future research on these important classes of nonlinear resonance, as well as spotlighting future research directions in the subfield. Moreover, two articles [25,26] pay special attention to the exploration of the impacts of structural confinements on SR, which to the best of our knowledge has been only been appreciated very recently, and for VR in a follow-up paper [34]. Comparative analysis presented here in the theme issue concludes that the VR-based strategies could provide better results in certain fields where SR is currently used, and it would be indeed very insightful to explore the VR phenomenon more rigorously in confined structures and energy harvesting devices where, hitherto, it has been given inadequate research attention.

## Data accessibility

No additional supporting data accompanied the submission.
